# Better Safe than Sorry: Prevention of Esophagojejunostomy Leak by Intraoperative Methylene Blue Test in Advanced Gastric Cancer

**DOI:** 10.1007/s11605-021-04921-6

**Published:** 2021-02-09

**Authors:** K. Sędłak, Karol Rawicz-Pruszyński, R. Mlak, J. Mielko, K. Gęca, W. P. Polkowski

**Affiliations:** 1 Department of Surgical Oncology, Medical University of Lublin, Radziwiłłowska 13 St., 20-080 Lublin, Poland; 2 Department of Human Physiology, Medical University of Lublin, Radziwiłłowska 11 St., 20-080 Lublin, Poland

**Keywords:** Gastric cancer, Esophagojejunostomy leak, HIPEC, Methylene blue

## Introduction

Total gastrectomy is a complex procedure with a high risk of complications.^1^ Although the percentage of complications after gastrectomy has recently decreased, the reoperation rate remains steady.^1^ Since the esophagojejunostomy leak (EJL) stays a critical postoperative complication in 5 to 14% of patients, it is essential to establish appropriate method of EJL prevention.^2^,^3^ Intraoperative methylene blue test (MBT) is one of few methods described so far and is potentially underestimated.^4^ The results of the MBT to check esophagojejunostomy (EJ) integrity suggest benefits, such as early recognition of EJL and possibility for immediate repair.^5^

The aim of this study was to verify the utility of intraoperative MBT in the prevention of the EJL after gastrectomy for advanced GC.

## Materials and Methods

One hundred fourteen consecutive patients with the esophagojejunostomy following total gastrectomy or proximal gastric resection and double-tract reconstruction (DTR) in whom the MBT was performed intraoperatively were suitable for analysis.

### Intraoperative Methylene Blue Test Technique

After completion end-to-side EJ, the integrity of anastomosis was tested by injection of methylene blue solution (2 ml of methylene blue dissolved in 100 ml of 0.9% NaCl) using a nasojejunal (NJ) tube at pressure of 20 kPa. NJ tube was placed proximal to anastomosis, and jejunum distal to anastomosis was clamped. Sterile gauze was used to cover anastomosis and reveal the potential site of a leak if present. The intraoperative leak was defined as the presence of administered dye solution on a gauze. If a solitary leak was found, additional stitches over the suture line were placed, and the test was repeated.

## Results

**Fig. 1:**
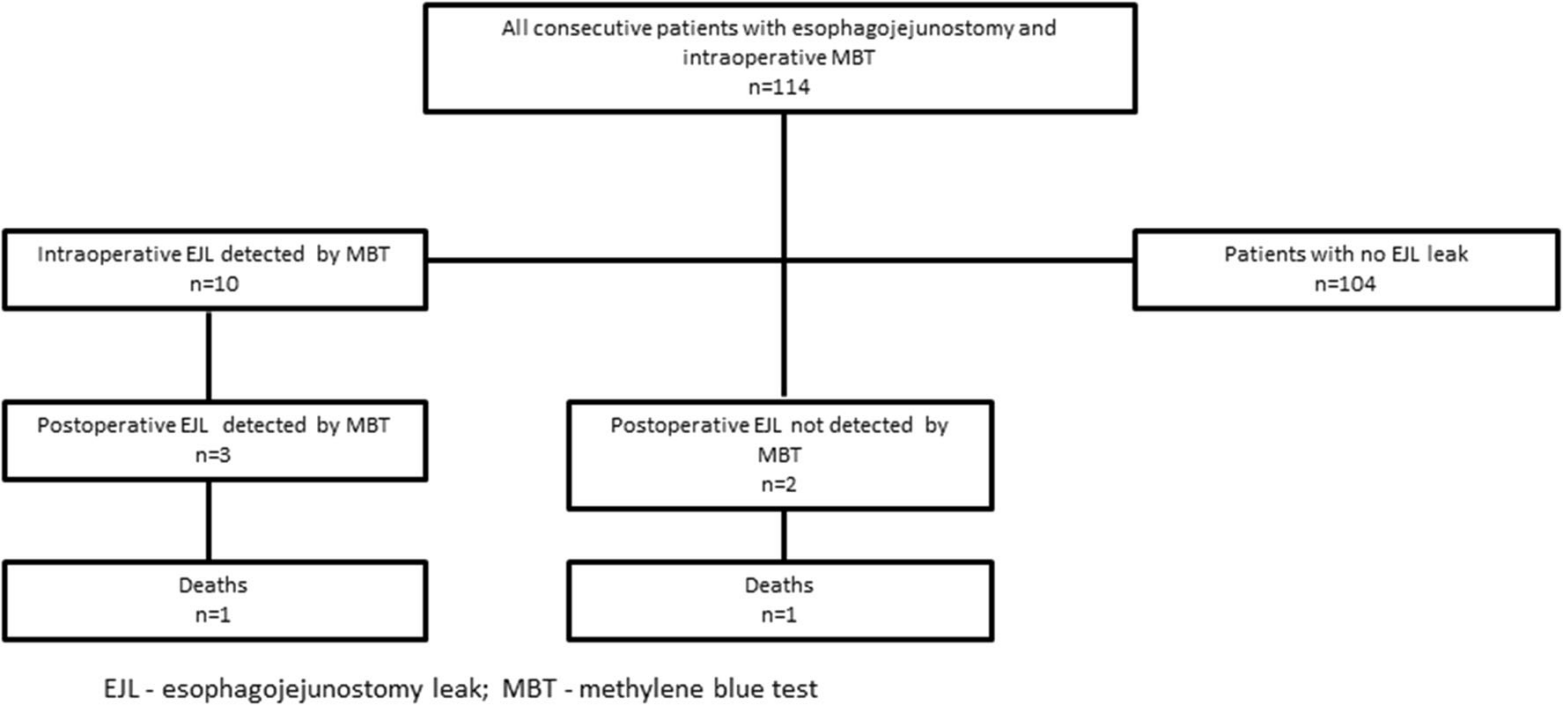
Algorithm of intraoperative and postoperative EJL. EJL. esophagojejunostomy leak; MBT. methylene blue test

The clinicopathological features of the 114 patients included in the study are shown in Table 1. The intraoperative leak was found in 10 (8.8%) patients. The postoperative leak was found in 5 (4.4%) patients. This means that in 5 cases, postoperative leak might have been prevented by MBT. Two patients with a postoperative leak died in the hospital: one among the leaks detected by intraoperative MBT (33.3%) and one among the postoperatively detected cases (50%). The anastomotic leak occurred most frequently in patients with pT3 tumors - two patients (40%) and pT4a tumors - two patients (40%). Longer hospitalization time was observed in patients with EJL (29 vs. 11 days; *p* = 0.0023). Similarly, significantly longer ICU stay was observed in patients with EJL (12 vs. 4 days; *p* = 0.0071). Sensitivity, specificity, positive predictive value (PPV), negative predictive value (NPV), and overall accuracy of the intraoperative MBT in the prediction of the postoperative, clinically apparent EJL were 60% (95%CI: 14.7–94.7%), 93.4% (95%CI: 87.2–97.4%), 30% (95%CI: 13.5–54.1%), 98.1% (95%CI: 94.6–99.3%), and 92.1% (95%CI: 85.5–96.3%) respectively. The algorithm of intraoperative and postoperative EJL is presented in Fig. 1.

**Table 1:** Clinicopathological variables of all patients included in the study

Variable	No. of patients *n* = 114 (%)*
Sex	
Male Female	67 (58.8%) 47 (41.2%)
Age (years)	
Average Standard deviation (±) Median (min–max)	57.9 12.5 58 (28–80)
Lauren histological type	
Intestinal Mixed Diffuse	42 (37.0%) 33 (28.7%) 39 (34.3%)
pT	
T0 T1a T1b T2 T3 T4a T4b	5 (4.5%) 1 (0.9%) 6 (5.4%) 17 (15.3%) 48 (42.3%) 23 (19.8%) 14 (11.7%)
pN	
N0 N1 N2 N3a N3b	45 (40.9%) 13 (11.8%) 19 (17.3%) 22 (20.0%) 11 (10.0%)
pM	
M0 M1	84 (73.7%) 30 (26.3%)
Neoadjuvant chemotherapy	
Yes No	81 (71.1%) 33 (28.9%)
Reconstruction method	
TG (Roux-en-Y) PG (DTR)	98 (86.0%) 16 (14.0%)
Intraoperative leak detected by MBT	
Yes No	10 (8.8%) 104 (91.2%)
Clinically apparent postoperative leak	
Yes No	5 (4.4%) 109 (95.6%)
CCI	
Average Standard deviation (±) Median (min–max)	17.4 26.1 0 (0–100)
Hospitalization time (days)	
Average Standard deviation (±) Median (min–max)	12.9 8.2 11 (4–59)
ICU hospitalization	
Yes No	21 (18%) 93 (82%)

*EJL* esophagojejunostomy leak, *CCI* comprehensive complication index, *ICU* intensive care unit, *TG* total gastrectomy, *PG (DTR)* proximal gastrectomy with double-tract reconstruction

## Discussion

The present study was undertaken to evaluate the utility of intraoperative MBT in the prevention of the EJL. Although the intraoperative MBT did not eliminate postoperative EJL, it might have reduced the number of postoperative clinical leaks by allowing the repair of the intraoperatively detected ones.

In recent report on perioperative complications from GC referral centers in 11 European countries belonging to the Gastrectomy Complications Consensus Group (GCCC),^6^ the most frequent surgical complication was anastomotic leak (9.8%). Authors suggest that portion of leaks may be linked to the employed surgical technique, calling for improvement in the learning strategy. It may be assumed that the reduced rate of EJL in our center (4.4%) was accomplished with a routine use of MBT. This study contains certain limitations: lack of postoperative upper gastrointestinal series, non-standardized definition of EJL leak, and, since there was no standardized way that the patients were tested for leak postoperatively, definitive conclusions about causation cannot be made.

## Conclusion

The MBT may reduce the amount of clinically apparent EJL.
